# 

**Published:** 1874-10

**Authors:** 


*Vol. XXVIII,* **OCTOBER, 1874.** *No. 10.*

THE

### DENTAL REGISTER,

A Monthly Journal Devoted to
the Interests of the Profession.

EDITED BY J. TAFT, D. D. S.

AND

PUBLISHED BY SPENCER, CROCKER & CO.,
OHIO DENTAL DEPOT.
168 Race Street, Cincinnati, Ohio.

TERMS: $2.50 Per Annum, in Advance; Single Copies 25c.

